# 595 Unremitting Pro-inflammatory Cell Phenotypes and Macrophage Activity, Following Paediatric Burn Injury

**DOI:** 10.1093/jbcr/irae036.229

**Published:** 2024-04-17

**Authors:** Donna Langley, Emma Krenske, Kate Zimmermann, Giorgio Stefanutti, Roy M Kimble, Andrew J A Holland, Mark Fear, Fiona M Wood, Tony Kenna, Leila Cuttle

**Affiliations:** QUT, Goodna, Queensland; Queensland University of Technology, Brisbane, Queensland; QUT, Brisbane, Queensland; Queensland Children's Hospital, Brisbane, Queensland; The University of Sydney, Westmead, New South Wales; University of Western Australia, Perth, Western Australia; Fiona Wood Foundation, Perth, Western Australia; Queensland University of Technology, Herston, Queensland; Queensland University of Technology, South Brisbane, Queensland; QUT, Goodna, Queensland; Queensland University of Technology, Brisbane, Queensland; QUT, Brisbane, Queensland; Queensland Children's Hospital, Brisbane, Queensland; The University of Sydney, Westmead, New South Wales; University of Western Australia, Perth, Western Australia; Fiona Wood Foundation, Perth, Western Australia; Queensland University of Technology, Herston, Queensland; Queensland University of Technology, South Brisbane, Queensland; QUT, Goodna, Queensland; Queensland University of Technology, Brisbane, Queensland; QUT, Brisbane, Queensland; Queensland Children's Hospital, Brisbane, Queensland; The University of Sydney, Westmead, New South Wales; University of Western Australia, Perth, Western Australia; Fiona Wood Foundation, Perth, Western Australia; Queensland University of Technology, Herston, Queensland; Queensland University of Technology, South Brisbane, Queensland; QUT, Goodna, Queensland; Queensland University of Technology, Brisbane, Queensland; QUT, Brisbane, Queensland; Queensland Children's Hospital, Brisbane, Queensland; The University of Sydney, Westmead, New South Wales; University of Western Australia, Perth, Western Australia; Fiona Wood Foundation, Perth, Western Australia; Queensland University of Technology, Herston, Queensland; Queensland University of Technology, South Brisbane, Queensland; QUT, Goodna, Queensland; Queensland University of Technology, Brisbane, Queensland; QUT, Brisbane, Queensland; Queensland Children's Hospital, Brisbane, Queensland; The University of Sydney, Westmead, New South Wales; University of Western Australia, Perth, Western Australia; Fiona Wood Foundation, Perth, Western Australia; Queensland University of Technology, Herston, Queensland; Queensland University of Technology, South Brisbane, Queensland; QUT, Goodna, Queensland; Queensland University of Technology, Brisbane, Queensland; QUT, Brisbane, Queensland; Queensland Children's Hospital, Brisbane, Queensland; The University of Sydney, Westmead, New South Wales; University of Western Australia, Perth, Western Australia; Fiona Wood Foundation, Perth, Western Australia; Queensland University of Technology, Herston, Queensland; Queensland University of Technology, South Brisbane, Queensland; QUT, Goodna, Queensland; Queensland University of Technology, Brisbane, Queensland; QUT, Brisbane, Queensland; Queensland Children's Hospital, Brisbane, Queensland; The University of Sydney, Westmead, New South Wales; University of Western Australia, Perth, Western Australia; Fiona Wood Foundation, Perth, Western Australia; Queensland University of Technology, Herston, Queensland; Queensland University of Technology, South Brisbane, Queensland; QUT, Goodna, Queensland; Queensland University of Technology, Brisbane, Queensland; QUT, Brisbane, Queensland; Queensland Children's Hospital, Brisbane, Queensland; The University of Sydney, Westmead, New South Wales; University of Western Australia, Perth, Western Australia; Fiona Wood Foundation, Perth, Western Australia; Queensland University of Technology, Herston, Queensland; Queensland University of Technology, South Brisbane, Queensland; QUT, Goodna, Queensland; Queensland University of Technology, Brisbane, Queensland; QUT, Brisbane, Queensland; Queensland Children's Hospital, Brisbane, Queensland; The University of Sydney, Westmead, New South Wales; University of Western Australia, Perth, Western Australia; Fiona Wood Foundation, Perth, Western Australia; Queensland University of Technology, Herston, Queensland; Queensland University of Technology, South Brisbane, Queensland; QUT, Goodna, Queensland; Queensland University of Technology, Brisbane, Queensland; QUT, Brisbane, Queensland; Queensland Children's Hospital, Brisbane, Queensland; The University of Sydney, Westmead, New South Wales; University of Western Australia, Perth, Western Australia; Fiona Wood Foundation, Perth, Western Australia; Queensland University of Technology, Herston, Queensland; Queensland University of Technology, South Brisbane, Queensland

## Abstract

**Introduction:**

Burns patients are susceptible to infection, and some studies suggest patients exhibit post-burn immunosuppression. However, the trajectory of immune cells after a burn injury are unknown. Furthermore, the immune cells associated with poor healing outcomes are also unknown. Aim 1 of this study characterised the immune profile of paediatric burn patients for over 18 months post-burn. Aim 2 identified the immune cells and inflammatory markers related to delayed burn wound healing.

**Methods:**

Flow cytometry was used to measure 26 cell lineage markers, chemokines and cytokines from both pro-inflammatory and anti-inflammatory immune profiles. In aim 1, Peripheral Blood Mononuclear Cells from 6 paediatric burn patients who had returned for burn and scar treatments over 4+ timepoints within 12 months post-burn were compared to 4 healthy controls. Aim 2 recruited 10 burn patients who re-epithelialized in < 21 days, 10 patients who re-epithelialized in >21 days, and 10 healthy control patient cells. All participants were children under 10 years age, with partial or full thickness scald or contact burns, 1-34% TBSA. Healthy controls were age and gender-matched.

**Results:**

While the level of basic immune cells such as CD4 and CD8 T cells remained relatively constant, over time T cells differentiated into proinflammatory cell phenotypes including Th17 and γδ-T cells. There was a 3-fold increase of IL-17 production from γδ-T cells in the first 3 weeks post-burn(p< 0.01). CCR4+CCR6+ T-regulatory cells positive were elevated at 9-18 months post-burn (p< 0.05). Alternatively activated (M2) macrophages displayed a 3.5-fold increase compared with controls (p< 0.01). Aim 2 preliminary results showed children with burns who re-epithelialized in under 21 days had variable abundance of T regulatory cells. Further, children with burns had a higher abundance of double positive CCR4+CCR6+ Tregs than healthy controls.

**Conclusions:**

Our results indicate immune cells do not increase or decrease over time post-burn, but they become highly specialised, inflammatory, and skin homing. These changes persist for 18 months post-burn, and this post-burn “immune distraction” may limit the ability of immune cells to prioritise responses to other threats, such as infections. Additionally, therapies which target the immune cells associated with delayed wound healing may enhance healing outcomes for paediatric burn patients.

**Applicability of Research to Practice:**

This project identifies targets in the immune/inflammatory response which if mediated, could improve burn wound healing outcomes.

